# Primary Care Antibiotic Prescribing and Infection-Related Hospitalisation

**DOI:** 10.3390/antibiotics12121685

**Published:** 2023-11-30

**Authors:** Stein Gerrit Paul Menting, Enya Redican, Jamie Murphy, Magda Bucholc

**Affiliations:** 1 School of Psychology, Ulster University, Coleraine BT52 1SA, UK; 2 School of Computing, Engineering and Intelligent Systems, Ulster University, Derry-Londonderry BT48 7JL, UK

**Keywords:** antibiotics, antimicrobial resistance, primary care, hospitalisation

## Abstract

Inappropriate prescribing of antibiotics has been widely recognised as a leading cause of antimicrobial resistance, which in turn has become one of the most significant threats to global health. Given that most antibiotic prescriptions are issued in primary care settings, investigating the associations between primary care prescribing of antibiotics and subsequent infection-related hospitalisations affords a valuable opportunity to understand the long-term health implications of primary care antibiotic intervention. A narrative review of the scientific literature studying associations between primary care antibiotic prescribing and subsequent infection-related hospitalisation was conducted. The Web of Science database was used to retrieve 252 potentially relevant studies, with 23 of these studies included in this review (stratified by patient age and infection type). The majority of studies (n = 18) were published in the United Kingdom, while the remainder were conducted in Germany, Spain, Denmark, New Zealand, and the United States. While some of the reviewed studies demonstrated that appropriate and timely antibiotic prescribing in primary care could help reduce the need for hospitalisation, excessive antibiotic prescribing can lead to antimicrobial resistance, subsequently increasing the risk of infection-related hospitalisation. Few studies reported no association between primary care antibiotic prescriptions and subsequent infection-related hospitalisation. Overall, the disparate results in the extant literature attest to the conflicting factors influencing the decision-making regarding antibiotic prescribing and highlight the necessity of adopting a more patient-focussed perspective in stewardship programmes and the need for increased use of rapid diagnostic testing in primary care.

## 1. Introduction

Antimicrobial resistance (AMR) has become one of the most serious global public health threats. In 2019, AMR contributed to an estimated 1.27 million deaths worldwide [1]. This number is predicted to rise to 10 million people per year by 2050, with a corresponding loss of $60–100 trillion in economic output [2]. The main driver of AMR is excessive and inappropriate prescribing of antibiotics, including prescribing antibiotics when they are not medically indicated (e.g., for viral illnesses) or when the choice of antibiotic (e.g., using powerful broad-spectrum antibiotics when a more targeted, narrow-spectrum antibiotic would be effective), dosage, or duration is not in line with established medical guidelines [3]. Both types of inappropriate prescribing could lead to a deterioration of the patient’s health and result in infection-related hospitalisation [4,5]. Excessive prescribing is relatively more common among children and the elderly [6], as these cohorts have higher perceived vulnerability and prognostic uncertainty, which often leads to ‘defensive prescribing’ by general practitioners (GPs) (i.e., a ‘treat, just in case’ approach) [7,8].

In the UK, it is estimated that the majority of all antibiotics (i.e., 79%) are prescribed in primary care settings by GPs [9]. Moreover, evidence suggests there may be considerable variation in the rates of prescribing between GP practices [6]. Studying infection-related hospitalisations after primary care antibiotic intervention may afford an opportunity to better understand the long-term health implications of antibiotic intervention and inform AMR prevention strategies. Consequently, the current narrative review aimed to investigate the relationship between antibiotic prescribing in primary care and the subsequent need for infection-related hospitalisation. As excessive antibiotic prescribing is relatively more common among children (<18 years old) and the elderly (>65 years old), specific attention was given to the prescription of antibiotics among these cohorts.

## 2. Results

**Figure 1:**
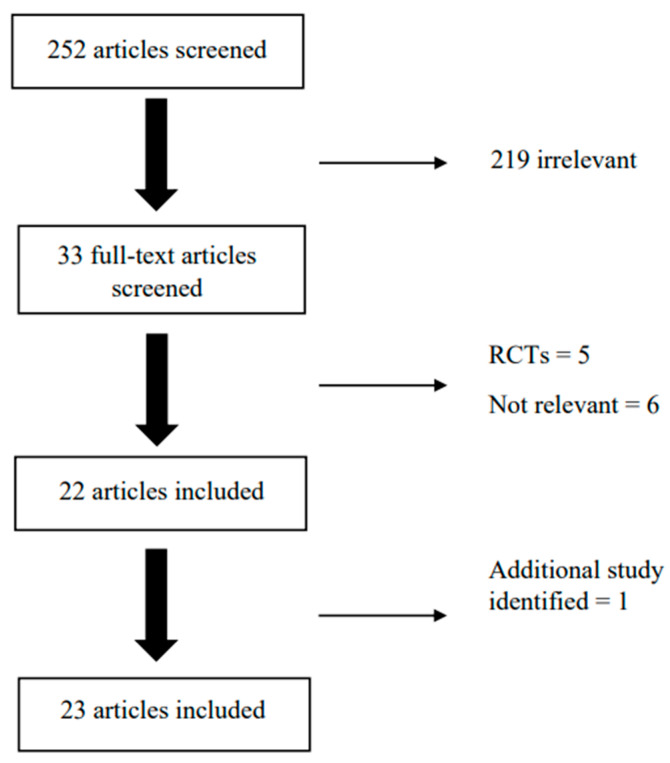
Study selection process.

The initial search of the scientific literature yielded 252 articles. After being independently screened by two reviewers, 33 articles were selected for full-text screening. Based on this screening, a total of 22 articles were deemed suitable. One additional study was identified through further searches, which resulted in a total of 23 articles (Figure 1). The majority (n = 18; 78.2%) of the studies were conducted in the UK. Regarding study design, 47.8% (n = 11) used a retrospective cohort design, 8.7% (n = 2) used a cross-sectional design, 8.7% (n = 2) used a cohort design, (n = 3) used an observational design, 4.3% (n = 1) used a case-control design, and 8.7% (n = 2) used a prospective cohort design. Of the included articles, six were conducted on child samples, seven on adult samples, four on elderly samples, and seven on samples containing all three age groups. Almost half of the studies were based on respiratory infections (RTIs) (n = 11; 47.8%), 30.4% (n = 7) of studies were based on urinary tract infections (UTIs), and 18.2% (n = 5) were based on multiple infection types. More detailed results will be presented in subcategories within these age groups and based on these types of infection. Table 1 includes information on study design, country, age group, infection type, and prescribed antibiotic. 

### 2.1. Children 

#### 2.1.1. Respiratory Infections

In young people, antibiotic treatment, administered early in the RTI course, reduced the probability of hospitalisation due to community-acquired pneumonia (CAP) and empyema [14]. Moreover, receiving antibiotics for empyema and parapneumonic effusion has been shown to reduce the likelihood of requiring surgical intervention by 43% compared to children who did not receive any pretreatment [13]. Compared to adults (i.e., aged 18–39 years), children are at greater risk of infection-related complications but not hospital admissions [31]. 

Although these studies highlighted the favourable aspects of antibiotic prescribing in primary care with respect to hospitalisation, other studies suggested differently [10,11,12,28]. Van Hecke and colleagues tested whether the number of antibiotic courses prescribed to 114,329 preschool children for acute RTIs in the preceding year was associated with the success of antibiotic treatment for recurrent RTIs [10]. Among the 1377 children who failed to respond to treatment, 1.2% (n = 103) required hospital admission. There was a clear dose–response relationship between antibiotic exposure and the likelihood of failing to respond to treatment, such that children who had received two or more courses of antibiotics in the preceding year were more likely to fail to respond to treatment. 

The timing of antibiotic prescriptions for RTIs in primary care, as well as their effects on hospitalisation, have been the focus of other studies. In particular, two large prospective cohort studies found that the time of prescribing (immediately or delayed) had no bearing on hospital outcomes [11,12]. Moreover, Winchester et al. showed that although prescribing antibiotics on the day of presentation at the GP was associated with a decreased risk of hospital admission for adults, this was not the case for children [28]. Finally, another study demonstrated the association between delayed antibiotic prescribing and a reduced likelihood of hospital admission in children compared to adults [27].

#### 2.1.2. UTIs

Ahmed et al., in their cross-sectional study of children presenting with *Escherichia coli* (*E. coli*)-positive UTIs, found that children admitted to hospitals were over two times more likely to have ampicillin-resistant urinary isolates compared to clinic patients [15]. Furthermore, the prescription of antibiotics within the previous six months was associated with resistance to trimethoprim/sulfamethoxazole, two of the main antibiotics used to treat bacterial infections.

#### 2.1.3. Multiple Infection Types 

Van Staa et al. investigated the association between frequent antibiotic use and the risk of infection-related hospital admission [32]. The most frequently prescribed antibiotic was amoxicillin across all quintiles of prior antibiotic use (35.1–52.5%), while phenoxymethylpenicillin was most common among those in the ‘lowest’ to ‘middle’ prior antibiotic use quintiles (11.0–15.0%) and trimethoprim was the most common in those in the ‘high’ and ‘highest’ quintiles (11.1–11.6%). In comparison to children in the highest quintile of prior antibiotic use, those in the lowest quintile of prior antibiotic use had a fives time lowest incidence rate ratio of infection-related hospitalisation. 

### 2.2. Adults

#### 2.2.1. Respiratory Infections 

Patients who received antibiotic treatment had an 8.16 lower overall risk of being hospitalised for CAP following an acute RTI as opposed to those who did not receive antibiotic treatment in a large administrative data-based study [29]. Moreover, studies looking at the timing of prescribing indicated that delayed antibiotic prescribing for upper respiratory tract infection (URTI) was associated with a 52% increase in infection-related hospital admissions [27], while prescribing on the day of diagnosis was associated with a decreased risk of lower respiratory tract infection (LRTI)-related hospital admission among patients aged 18 to 64 years [28]. Although Van Staa et al. found that the adverse consequences of delayed antibiotic prescribing were highest among adults and lowest among children, patients’ predicted risk of infection-related hospital admission was not linked to delayed antibiotic prescribing [27].

Two other studies reported no impact of primary care antibiotic prescribing on subsequent hospitalisation. Loffler et al. used a large RCT database of individuals in Germany with acute respiratory tract infection (ARTI) and discovered that neither the patient’s antibiotic status nor the doctor’s individual antibiotic prescription rates for ARTI had a significant association with hospitalisation [16]. Similarly, in an observational study of 28,779 patients with LRTI, it was found that neither immediate nor delayed antibiotics were associated with a significant reduction in subsequent hospital admission or death. However, delayed prescribing was associated with a 36% reduction in nonresolving or new symptoms [17]. 

#### 2.2.2. UTIs

Compared to respiratory infections, where outcomes of antibiotic prescribing in primary care were largely favourable, findings with respect to UTIs indicated antimicrobial resistance to represent an issue. Specifically, a study conducted on a small sample of adults affected by UTI (n = 80) found that resistance to trimethoprim, a common antibiotic used to treat bladder infections, was evident in adults who had received an antibiotic prescription in the preceding year [21]. Two studies using much larger administrative datasets reported similar findings [19,20]. Specifically, one study looked at Danish adults with community-acquired UTIs with *E. coli*, both those producing extended-spectrum beta-lactamase (ESBL) and those not [20]. Findings from this study demonstrated how significant correlates of treatment failure (i.e., new prescription of antibiotics for UTIs or admission to hospital due to UTI at 14 and 30 days, respectively) included ESBL production, older age, male sex, and resistance to empirical antibiotics [20]. Compared to the other studied antibiotics, pivmecillinam demonstrated the lowest rate of treatment failure. Another study based on individuals aged 16 years or older in the UK found that prior antibiotics were associated with a dose-dependent increase in the odds of urinary infection-related hospital admission [19]. Notably, not receiving antibiotic treatment within seven days was linked to lower risks of urinary infection-related hospital admission, which, according to the authors, likely reflected the fact that untreated patients were younger, had fewer comorbid conditions, and had less prior exposure to antibiotics than treated patients [19]. 

#### 2.2.3. Multiple Infection Types 

Findings from studies investigating multiple infection types are largely heterogenous. Van Bodegraven et al. found that the prevalence of infection-related hospital admissions was negatively associated with practice-level antibiotic prescribing; specifically, a 10.4% increase in antibiotic prescribing was associated with a 5.7% decrease in the frequency of infection-related hospital admissions [31]. The magnitude of these associations varied according to infection type, with LRTIs showing the greatest reduction in infection-related hospital admissions, followed by UTIs and URTIs. These associations were additionally affected by the age of the patients, with the effects being largest for those between the ages of 18 and 39 and weakest for those who were younger or older. Moreover, in a study assessing the impact of a national antimicrobial stewardship programme, there was evidence, albeit indirect, that reduced antibiotic prescribing in primary care did not result in increased hospital admissions [22]. Conversely, a large GP-based study showed how a higher rate of antibiotic prescribing in primary care was linked to a significantly higher number of adjusted hospital admissions due to complications from RTIs and UTIs regardless of the type of antibiotic prescribed or the age or postgraduate training or appointment (permanent or nonpermanent) of the GP [18]. Another study by Van Staa et al. demonstrated how the risk of infection-related hospital admissions was highest in patients with frequent prior antibiotic use [32]. Finally, a study by Mistry et al. using administrative data found no association between antibiotic prescribing in primary care and the risk of infection-related hospital admission for URTI, LRTI, or UTI [30]. However, findings demonstrated how the hazard ratios regarding the incidence of hospital admission due to infection-related complications were highest for the youngest patients (<5 years old) and the oldest patients (80+ years old). 

### 2.3. Elderly

#### 2.3.1. Respiratory Infections

A large-scale study followed 19.6 million patients for 30 days after consultation with a GP for one of the various common infections (49% URTI, 12% LRTI, 8% UTI) [31]. It was reported that the GPs’ antibiotic prescription rate was not associated with the odds of infection-related complications or hospital admissions in patients over 60 years old. Likewise, among patients who were 65 years and older, receiving an antibiotic prescription on the date of LRTI diagnosis was not associated with hospitalisation for pneumonia or another LRTI in the following 30 days, although it did decrease the odds of respiratory-infection-related mortality [28]. These studies suggest that antibiotic prescription as a response to a respiratory infection may not specifically prevent infection-related hospitalisation. Conversely, Van Staa et al. demonstrated that a delay in the prescription of antibiotics in response to a URTI diagnosis increased the odds of infection-related hospitalisations in the following 30 days in elderly patients (>60 years old) [27]. Furthermore, in a cohort of 39,211 elderly patients, the risk of all-cause hospitalisation decreased when antibiotics were prescribed within 28 days of the community-acquired pneumonia diagnosis, even when controlling for a wide range of demographic factors, comorbidities, frailty factors, medications, and vaccinations [23].

#### 2.3.2. UTIs

In a population-based cohort study, 157,264 elderly patients were followed for 60 days after an initial lower UTI diagnosis by a GP [25]. The odds of hospitalisation for bloodstream infection (incl. sepsis), the length of stay in the hospital, and all-cause mortality were significantly lower in patients that were immediately prescribed antibiotics at initial diagnosis compared to those who were prescribed antibiotics in the seven days after initial diagnosis or those who were not prescribed antibiotics at all. Although there was no difference in the odds of bloodstream infection following an immediate prescription of the two most prescribed antibiotics (*p* = 0.41), an immediate trimethoprim prescription did reduce the 60-day survival rate (98.5%), compared to nitrofurantoin (98.7%, *p* < 0.001). In contrast to this study, when Shallcross et al. examined the same population, it was reported that urosepsis was only listed as the main reason for hospital admission in 10.3% of all bloodstream infection cases [24]. Furthermore, after adjusting for patient demographics, year of consultation, comorbidities, smoking status, recent hospitalisations, recent Accident and Emergency attendances, recent antibiotic prescribing, and home visits, there was no evidence that delaying or withholding antibiotic prescription was associated with an increased risk of bloodstream infection in the 60 days following a UTI diagnosis. However, an immediate antibiotic prescription was associated with a decrease in all-cause mortality, as well as hospitalisation for conditions unrelated to a bloodstream infection or UTI. When studying the effectiveness of immediate antibiotic prescription after UTI diagnosis, it was reported that older age increased the likelihood of treatment failure, characterised by a new prescription of antibiotics or hospitalisation [20]. Likewise, in a population of elderly patients, 6% of patients were prescribed another antibiotic within 14 days of the initial UTI, pointing towards a treatment nonresponse, possibly due to antimicrobial resistance [26]. Within this group, nitrofurantoin prescription was associated with reduced odds of hospitalisation compared to trimethoprim.

## 3. Discussion 

This review describes the existing evidence base regarding the association between primary care prescribing of antibiotics and later infection-related hospitalisations. Results show that, regardless of age group or infection type, antibiotic prescribing in primary care has been associated with both higher and reduced frequency of infection-related hospitalisations. Notably, however, a history of repeated antibiotic prescriptions was consistently shown to be associated with an increased risk of infection-related hospitalisation across studies.

The prevalence of paediatric infections that are resistant to antibiotics is rising worldwide [33]. RTIs are particularly common in children, accounting for approximately 74% of all paediatric antibiotic prescriptions in primary care [34]. Notably, randomized control studies have shown that antibiotics are ineffective for several RTIs, which is why they are typically not recommended for the treatment of most RTIs [35]. Although clinicians often prescribe antibiotics to children when there is a clear indication, it is often also used as a precautionary measure (i.e., “just in case”) and because of parental pressure [7]. This is not necessarily negative, given that some studies show that the prescribing of antibiotics to children for RTIs in primary care can be beneficial in terms of lowering hospital admissions or the need for surgical intervention [13,14,27,31]. Moreover, others indicate that it has no meaningful impact on hospitalisation risk [11,12,28]. However, children represent one of the most vulnerable populations to infections with antibiotic-resistant bacteria [36], as reflected in one study where repeated prescribing of antibiotics for RTIs increased the risk of infection-related hospitalisation [10]. This study emphasised that repeated antibiotic use increased the likelihood of bacterial resistance in children and, as a result, primary care physicians should pay special attention to avoid unnecessary antibiotic prescriptions. It was notable that the findings varied as they did across studies. This was likely attributable to the variation in sample sizes (some studies included 200 children, while others included more than 114,329 children), the different age ranges included (one study focussed on preschoolers only, while others focussed on 0 to 14-year-olds and 6-month-olds to 16-year-olds, respectively), the different study designs (some studies used a case-control design, others used large administrative databases), as well as the different types of RTIs under investigation (some studies looked at pneumonia, while others looked at RTIs more broadly). 

Ahmed et al. was the only included study specifically investigating the relation between antibiotic prescribing in primary care and hospitalisation for UTIs in children [15]. It was reported that the risk of developing a resistance to antibiotics used to treat UTIs was relatively higher in children who had just received an antibiotic prescription, specifically for trimethoprim and sulfamethoxazole. These findings are consistent with those identified by Duffy et al., who demonstrated that previous trimethoprim prescription increased the risk of trimethoprim-resistant *E. coli* in the urine samples of children (<16 y.o.) with a suspected UTI diagnosis, with more recent prescriptions resulting in an increased risk of resistance [37]. Additionally, Gruneberg and Shaw also demonstrated increased antibiotic resistance in children with UTIs who had previously been prescribed a sulphonamide [38].

Inappropriate antibiotic prescribing tends to be most pervasive in the adult population [39]. In contrast to the child studies, adult studies demonstrated mostly favourable impacts of antibiotic prescribing in primary care for RTIs. Specifically, antibiotic prescribing for RTIs in primary care was linked to decreased risk of hospitalisation due to CAP for patients with an acute RTI [29], while immediate prescribing was linked to a lower risk of hospitalisation [27,28]. From these findings, it would seem that the benefit of prescribing outweighs the harmful effects associated with antibiotic prescribing practices for adult RTIs in primary care. However, it has also been demonstrated that RTIs are usually self-limiting and improve without specific treatment [17]. This should be taken into account, given that repeated antibiotic prescribing in primary care has been shown to increase the risk of antimicrobial resistance [5], hospitalisation for infection-related complications [32], and mortality [4]. 

In the case of UTIs in adults, resistance to antibiotics that cater to bladder infections was found to represent a major issue with respect to hospitalisation [19,20,21]. Prior research has shown how exposure to trimethoprim is associated with trimethoprim-resistant urinary tract infection [40,41], while consumption of amoxicillin is associated with an increased risk of ampicillin-resistant *E. coli* infection [42]. Overall, it is a major cause for concern that gram-negative bacteria, the type of bacteria that frequently cause UTIs, are becoming more resistant to antibiotics, reducing the range of possible treatments for UTI patients [43].

The studies in which multiple infection types were explored simultaneously generally reported conflicting findings. Some studies indicated that antibiotic prescribing in primary care was associated with a reduction or no increase in hospitalisation [22,31], whereas one study reported higher rates of hospitalisation [18], and yet another reported no association between antibiotic prescribing in primary care and antibiotic resistance [30]. It is unlikely that the examination of multiple infection types explains these different findings, given that most of the studies focussed only on RTIs and UTIs [18,30,31]. However, different study designs were adopted such that the target population of the Urrusuno et al. study was GPs, whereas other studies focussed on large representative samples of patients using administrative data [18]. 

Akin to children, the rate of infection-related diagnosis in primary care and hospitalisation is higher in the elderly [19,44]. Yet, antibiotic prescription is complicated in this population due to the fact that this group is generally more likely to experience frailty, comorbidities, polypharmacy, and antimicrobial resistance [45]. Frailty and comorbidities have been reported to increase the odds of both infection-related and all-cause hospitalisation after a diagnosis of infection in primary care [23,24,25]. For example, a decrease in renal function increases the risk of infection-related hospitalisation in elderly patients [26]. Given the prevalence of frailty and comorbidities in elderly patients, an argument could be made that in this specific cohort, the focus should be shifted away from preventing specifically infection-related hospitalisation and more towards all-cause hospitalisation and mortality. Taking this into consideration, the included studies in this review conclude that immediate antibiotic prescription is favourable, as it reduces the odds of all-cause hospitalisation and mortality [23,24]. However, before proclaiming immediate antibiotic prescription as the course of action after diagnosing an infection, one should consider the adverse effects of antibiotic prescribing throughout a lifetime. Older age was associated with a relatively higher rate of additional antibiotic prescriptions after the initial prescription [20,26,32], pointing towards increased antibiotic resistance and the associated risk factors for the patient. Additionally, the rate of polypharmacy is known to be higher in later life [46]. Immediate antibiotic prescribing could interfere with medication already taken by the patient, possibly resulting in adverse drug effects [47].

### 3.1. Antibiotic Prescribing in Primary Care

The heterogenous findings of the current review could explain some of the variability in prescription rates between primary caretakers [6]. Further complicating the matter of antibiotic prescribing for practitioners in primary care is the need to find a balance between combatting antimicrobial resistance on a population level and preserving the health and wellbeing of their individual patients [48]. Antimicrobial stewardship programmes recommend limiting the use of new and broad-spectrum antimicrobials or leaving milder, mostly self-limiting infections untreated [49]. Although most practitioners acknowledge the need for such programmes, their antibiotic prescribing habits are chiefly informed by patient-specific issues, such as efficacy, cost, and tolerability [48,50]. The use of (broad-scale) antibiotics is characterized by practitioners as having large short-term benefits with low short-term risks, therefore providing a satisfying solution for the immediate, pressing, and visible problems presented by patients in a day-to-day setting [51]. We should also consider patients’ ignorance towards the difference between types of infections (‘germs are germs’) and potential side effects of antibiotic use (e.g., influence on gut flora). Furthermore, the patients’ first concern is improving their own health before considering potential risks to society [50]. These phenomena are even more prevalent in developing countries [52,53]. Given the above, efforts to change practitioners’ prescribing behaviour might benefit from shifting the narrative away from antimicrobial resistance as an abstract, global problem [52]. Instead, the emphasis could be put on the fact that inappropriate antibiotic prescribing on an individual level could lead to antimicrobial resistance in the patient [5] and the associated increased risk of prolonged hospital stay and mortality for that individual [4,54]. Furthermore, there are strong regional effects of differences in antimicrobial use and resistance, which suggest that reducing antimicrobial use can benefit a region or nation even if its neighbours adopt a less effective programme [49]. 

Another way that antimicrobial stewardship programmes can improve the treatment of infectious diseases is through rapid microbiological tests [55]. Rapid diagnostic tests can help slow AMR, reduce unnecessary antibiotic administration, and preserve the efficacy of currently prescribed antibiotics [2]. Although it is outside the scope of this review to discuss the wide range of rapid diagnostic tests that are in existence, there is an extensive evidence base documenting their efficacy [55,56,57,58]. Rapid diagnostic tests have been found to increase diagnostic accuracy, shorten hospital stays, and lower both mortality and healthcare costs when used in conjunction with antimicrobial stewardship [55]. However, there are several barriers that hinder the use of such tests, such as GPs’ tendency to rely more on their own clinical judgement than the findings of a rapid test, patients’ demands for antibiotics, the patient–provider relationship, and a lack of provider education or evidence regarding some rapid diagnostic tests and their proper application [59]. As a result, there is a need for implementation techniques that are multifaceted and address discrepancies between knowledge and behaviour, such as patient education and customized approaches for each rapid diagnostic test within different departments [59].

### 3.2. Limitations

The studies included in this review were all based on populations in developed countries, with the majority of included studies being based on a UK population. Caution should be taken when transferring the findings of this review to other populations, as there will likely be differences in population characteristics, healthcare systems, and the public attitude towards antibiotic prescribing. Additionally, given the regional nature of antimicrobial resistance, it is key that the effect of antibiotic prescribing in primary care on hospitalisation is studied in countries beyond the UK. Furthermore, a considerable number of included studies analysed administrative data or were designed as retrospective cohort studies. Although these quantitative studies provide excellent insight into population-based phenomena, they often rely on data that were initially not collected with a research purpose in mind. For example, although a GP’s antibiotic prescription rate can be tracked through (electronic) health records, these do not include factors such as the severity of clinical presentation, the patient’s prior medical history, or the patient’s social circumstances [24]. In a similar vein, the inability to control the variables that may be investigated is another significant drawback of retrospective cohort designs [60]. For instance, and as previously highlighted, there are likely to be a plethora of factors that influence the association between prescribing patterns in primary care and infection-related hospitalisations. This was observed in one of the studies where they were unable to evaluate factors that could have an impact, such as the standard of care, accessibility to general practitioners and their practices, the availability of consultations, and the prescribing preferences of physicians [31]. Moreover, other potential limitations of retrospective cohort designs include the possibility of confounding by indication, misclassification biases, the variability in coding and recording information across different medical practices, and missing data [25]. The findings of the quantitative studies included in this review should, therefore, ideally be combined with results from qualitative research, which could provide additional insight into the specifics of GPs’ antibiotic prescribing habits [61], as well as account for a range of potential confounding variables. 

## 4. Materials and Methods

Two reviewers used the Web of Science database to conduct searches. The keywords and Boolean operators that were employed included (“antibiotic prescr*” or “antimicrobial prescr*”) and (“primary care”) and (“hospital*” or “secondary care”) and (“urinary tract infection*” or “UTI” or “sepsis” or “pneumonia” or “respiratory tract*” or “Septicaemia”). Only English-language articles published between 2009 and 2023 were included. Additional searches were conducted on Google Scholar and PubMed for other potentially relevant articles. Both reviewers independently screened the title and abstracts of all articles retrieved. After selecting those that were deemed suitable for full-text screening, both reviewers screened all articles and discussed their eligibility for inclusion. 

## 5. Conclusions

The growing occurrence of pathogens resistant to antimicrobials poses a major threat to public health. The findings of this review attest to the considerable heterogeneity in the effect of antibiotic prescribing in primary care on the risk of subsequent hospitalisation. However, there is consistent evidence that a history of repeated antibiotic use in the same patient increases the risk of infection-related hospitalisation. It would seem that although antibiotic prescription in primary care could reduce the risk of hospitalisation, inappropriate or excessive prescribing can have negative consequences, including antibiotic resistance in both an individual patient and at the population level. It may be advantageous to place greater emphasis on integrating diagnostic testing along with stewardship programmes into all primary care settings, given the difficult task general practitioners face in balancing the needs of their own patients with those of the larger population. Going forward, it will be necessary that studies investigating the association between antibiotic prescribing in primary care and the risk of infection-related hospitalisation also account for the repeated prescribing of antibiotics. Moreover, it is necessary that comparable research be conducted in low- and middle-income nations, as it is only then that international policies to enhance prescribing practices in primary care can be developed. 

## Figures and Tables

**Table 1:** A summary of the design of the included studies.

Study	Design	Age Group	Country	Infection Type	Prescribed Antibiotic (% of Sample Total)
Van Hecke et al. (2019) [10]	Observational cohort	Children	UK	Respiratory (ARTI)	Penicillin (76.7) (amoxicillin, co-amoxiclav) Penicillin V (12.0) Macrolides (10.1)
Redmond et al. (2018) [11]	Prospective cohort study	Children	UK	Respiratory (Acute cough and RTI)	
Hay et al. (2016) [12]	Prognostic cohort study	Children	UK	Respiratory (Acute cough and RTI)	
Mahon et al. (2016) [13]	Retrospective cohort	Children	New Zealand	Respiratory (Empyema and parapneumonic effusion)	
Crocker et al. (2012) [14]	Case-control study	Children	UK	Respiratory (Pneumonia)	
Ahmed et al. (2015) [15]	Cross-sectional	Children	USA	UTI	Ampicillin, Ciprofloxacin, Ceftriaxone, Cefazolin, Ampicillin/sulbactam, Ceftazidime, Nitrofurantoin, Gentamycin, Levofloxacin, Tobramycin, Trimethoprim/Sulfamethoxazole
Loffler et al. (2020) [16]	RCT-based database	Adults (18+)	Germany	Respiratory (ARTI)	
Little et al. (2014) [17]	Observational Study	Adults (16+)	UK	Respiratory (LRTI)	
Urrusuno et al. (2018) [18]	Cross-sectional	Adults (18+)	Spain	RTI and UTI	Amoxycillin/clavulanate, amoxycillin, cefuroxime, ciprofloxacin, clarithromycin
Aryee et al. 2023 [19]	Retrospective cohort	Adults	UK	UTI	Nitrofurantoin, trimethoprim, fosfomycin, pivmecillinam
Jansåker et al. (2019) [20]	Retrospective cohort	Adults	Denmark	UTI (Complicated and uncomplicated)	Pivmecillinam (68.7)
					Sulfamethizole (22.8)
					Nitrofurantoin (2.9)
					Ciprofloxacin (2.3)
					Trimethoprim (2.1)
					Aminopenicillins (1.2)
Costelloe et al. (2014) [21]	Retrospective cohort	Adults	UK	UTI	Trimethoprim (20)
					Nitrofurantoin (18)
					Amoxicillin (13)
					Ciprofloxacin (11)
					Co-amoxiclav (10)
					Erythromycin (10)
					Flucloxacillin (8)
					Clarithromycin (3)
					Other (7)
Balinskaite et al. (2019) [22]	Retrospective cohort	Adults	UK	Various	
Millett et al. (2015) [23]	Cohort study	Older adults	UK	Respiratory (Pneumonia)	
Shallcross et al. (2020) [24]	Retrospective cohort	Older adults	UK	UTI	
Gharbi et al. (2019) [25]	Retrospective cohort	Older adults	UK	UTI	Trimethoprim (54.7)
					Nitrofurantoin (19.1)
					Cephalosporins 911.5)
					Amoxicillin (9.5)
					Quinolones (4.4)
					Pivmecillinam (0.4)
Ahmed et al. (2018) [26]	Retrospective cohort	Older adults	UK	UTI	Trimethoprim (60.6)
					Nitrofurantoin (20.7)
					Cefalexin (6.2)
					Amoxicillin (4.5)
					Co-amoxiclav (4.5)
					Ciprofloxacin (3.2)
Van Staa et al. (2021) [27]	Cohort study	All	UK	Respiratory (UTRI)	Amoxicillin, clarithromycin, doxycycline, erythromycin, phenoxymethylpenicillin
Winchester et al. (2009) [28]	Observational study	All	UK	Respiratory (LRTI)	Penicillins (72.8)
					Macrolides (15.5)
Meropol et al. (2013) [29]	Retrospective cohort	All	UK	Respiratory (Acute nonspecific respiratory infections)	Penicillins (68)
					Macrolides (13)
					Cephalosporins, cephamycins, and other β-lactams (7)
					Tetracyclines (7)
					Sulphonamides and
					trimethoprim (3)
					Quinolones (1)
Mistry et al. (2020) [30]	Retrospective cohort	All	UK	URTI, LRTI, or UTI	
Van Bodegraven et al. (2021) [31]	Retrospective cohort study	All	UK	Various	
Van Staa et al. (2020) [32]	Cohort study	All	UK	Multiple	Amoxicillin (35.1–52.5)
					Phenoxymethylpenicillin (4.1–15.0)
					Trimethoprim (9.8–11.6)
					Erythromycin (7.0–9.0)
					Clarithromycin (4.0–7.5)
					Cefalexin (3.3–9.0)
					Doxycycline (2.4–5.5)
					Nitrofurantoin (1.6–6.8)
					Flucloxacillin (1.5–1.9)
					Ciprofloxacin (1.0–5.7)
					Cefaclor (0.5–1.3)

Note: RTI = respiratory tract infection, UTI = urinary tract infection, ARTI = acute respiratory tract infection, LRTI = lower respiratory tract infection, UTRI = upper respiratory tract infection.

## Data Availability

Data sharing not applicable.
